# Hemophagocytic lymphohistiocytosis secondary to histoplasmosis: A case report in a patient with AIDS and recent SARS-CoV-2 infection and minireview

**DOI:** 10.1016/j.heliyon.2023.e18537

**Published:** 2023-07-21

**Authors:** Luca Pipitò, Alice Annalisa Medaglia, Marcello Trizzino, Alessandro Mancuso, Bianca Catania, Salvatrice Mancuso, Cinzia Calà, Ada Maria Florena, Antonio Cascio

**Affiliations:** a Department of Health Promotion, Mother and Child Care, Internal Medicine and Medical Specialties “G D'Alessandro,” University of Palermo, Palermo, Italy; b Infectious and Tropical Disease Unit and Sicilian Regional Reference Center for the Fight Against AIDS, AOU Policlinico “P. Giaccone”, 90127 Palermo, Italy; c Palermo Fast-Track City, Casa dei Diritti, Via Libertà 45, 90143 Palermo, Italy; d Microbiology and Virology Unit- Department of Health Promotion, Mother and Child Care, Internal Medicine and Medical Specialties “G D'Alessandro,” University of Palermo, Palermo, Italy; e Unit of Pathology, Universital Hospital Paolo Giaccone, Palermo (PA), viale del vespro 147, Palermo, Italy

**Keywords:** Histoplasmosis, AIDS, HIV, Hemophagocytic lymphohistiocytosis

## Abstract

Here, we describe the case of a naïve HIV late presenter female African patient with progressive disseminated histoplasmosis and a severe life-threatening clinical picture in a non-endemic area. She had not visited Africa in the past decade. She developed a reactive hemophagocytic lymphohistiocytosis and an acute psychiatric disorder. Histoplasmosis was diagnosed after two bone marrow biopsies. Therapy with liposomal amphotericin B resulted in rapid and progressive improvements in blood examinations and clinical conditions, including the disappearance of psychiatric disorders. The characteristics of our case were compared with those of all other cases of hemophagocytic syndrome secondary to histoplasmosis in HIV-positive patients reported in PubMed. In conclusion, clinicians outside endemic areas should evaluate histoplasmosis as a cause of severe clinical picture, especially in a patient with a travel history to an endemic area, even after many years, considering the possible reactivation of latent infection.

## 1 Introduction

*Histoplasma capsulatum* is the etiological agent of histoplasmosis and a cause of severe infection in patients with impaired immune systems, especially those with Acquired Immune Deficiency Syndrome (AIDS). It is a dimorphic fungus [1]^,^ and the transition from the mycelial to the yeast phase is the most important factor for its pathogenicity in humans. The cell-mediated response is essential for clearance of the fungus, and the hallmark of the tissue response is the development of caseating or non-caseating granulomas, which are absent in cases of immunodeficiency [2].

Histoplasmosis has been reported in every continent. It is acquired through inhalation of conidia from the filamentous phase of the fungus present in soil and bird and bat droppings and can cause potentially lethal infection [3,4]. Disruptions to the soil due to excavation or construction can release infectious particles, and spelunkers, builders, climbers, and farmers are at the highest risk of infection. Acute pulmonary disease may be asymptomatic and self-recovering or characterized by symptoms such as cough, chest pain, headache, malaise, arthromyalgia, and a high fever associated with radiological findings of patchy pneumonia with areas of interstitial infiltrate and hilar lymphadenopathy, and in some cases, cavitary lesions, especially in chronic cases. In AIDS patients, severe progressive disseminated histoplasmosis is characterized by hepatosplenomegaly, lymphadenopathy, cutaneous lesions (maculopapular, petechiae, ecchymosis, and pustules), and rarely, meningitis and encephalitis [2,[4], [5], [6]]. In this category, untreated infections resulted in 100% mortality [4]. Reactivation of the latent disease may occur in immunosuppressed populations [5].

Furthermore, the culture of *H. capsulatum* is highly specific but has limitations. Specifically, the fungus may take several weeks to grow, and high-level laboratory infrastructure is required for culture handling. Specific antigen detection in serum or urine is the mainstay of diagnosis [2,5]^,^ and galactomannan [7] and 1–3 Beta-d-glucan [8] can positively support this diagnosis. Cytological and histological methods are essential to confirm the diagnosis [9].

Here, we present the case of a naïve HIV African woman with a recent SARS-CoV-2 infection, the onset of pancytopenia, and psychiatric disorders, in whom histoplasmosis-related reactive hemophagocytic syndrome (HLH) was diagnosed on a microscopic basis due to the positivity of serum galactomannan and a favorable response to a specific therapy.

## 2 Methods

A PubMed literature search combining the words «(hemophag * OR hemophag* OR lymphohistiocytosis) AND Histoplas*[TITL] ».

Data on age, sex, nationality, human immunodeficiency virus (HIV) viral load, CD4 count, symptoms, clinical picture, criteria for reactive HLH, microbiological findings, treatment, and outcomes were collected and subsequently analyzed to evaluate their associations with mortality. Continuous variables are summarized as mean ± standard deviation or median and interquartile range (IQR), whereas categorical variables are presented as absolute and relative frequencies. Differences in means were evaluated using the Mann-Whitney *U* test, and the χ2 test was applied to categorical variables. Statistical significance was set at a p-value <0.05. Crude odds ratios (cORs) and 95% confidence intervals (CI) for the association between mortality and potential risk factors were calculated using univariate analysis. Only the factors associated with mortality were included in the multivariate analysis. Only statistically significant results are reported.

## 3 Case report

A 54-year-old female from Ghana, living in Italy for many years without a significant medical history, presented to the Emergency Department with syncope and persistent fever for a few days. She had not visited Africa in the past decade. On physical examination, the patient had a temperature of 38.5 °C, pulse rate of 110/min, respiratory rate of 20/min, blood pressure of 100/60 mmHg, and 96% oxygen saturation. She had ecchymosis-like lesions on her lower limbs and postural instability, such as ataxia.

Laboratory examination revealed pancytopenia: hemoglobin, 9.1 g/dl; platelet count, 112 × 10^3^/μL; leukocyte count, 2,74 × 10^3^/μL. The C-reactive protein (CRP) level was 80.4 (<5) mg/L, and the procalcitonin (PCT) level was 5.5 (<0.05) μg/L. She had mildly increased aspartate aminotransferase (AST) (52 U/L), alanine aminotransferase (ALT) (17 U/L), and glutamyl transpeptidase (GGT) levels (47 U/L). Furthermore, albumin was 33.0 g/L. The patient's kidney function was normal. Serum ferritin was elevated (2,997 ng/ml), and the Beta2-Microglobulin was 5.3 (range between 0.2 and 0.8) mg/L. Triglycerides were 242 mg/dl, and cholesterol levels were within the normal range. Fibrinogen and protein electrophoresis results were unremarkable, and D-dimer levels were elevated at 21,790 ng/ml (range, 0–600 ng/ml).

A nasopharyngeal swab was positive for SARS-CoV-2 and negative after three days. The HIV screening test result was positive, and the patient was transferred to the Infectious Disease Department. On admission, total body computed tomography (CT) showed splenomegaly, abdominal lymphadenopathy (∅ 1.6 cm), and ventricular system of brain expansion due to cerebral atrophy. The basal HIV viral load and CD4 cell count were 1,270,000 copies/ml and 25 cells/mm^3^ (6%; CD4/CD8 = 0.1), respectively.

Screening for opportunistic infections, including *Cryptococcus neoformans* serum antigen, *Pneumocystis jirovecii* polymerase chain reaction of induced sputum, *Mycobacterium tuberculosis* complex microscopy, and induced sputum polymerase chain reaction yielded negative results. Immunoglobulin G (IgG) antibodies against *Toxoplasma gondii* tested positive. Screening for syphilis and hepatotropic viruses yielded negative results. Low-level viremia positivity for Cytomegalovirus (CMV) and Epstein Barr virus (EBV) DNA was detected. Leishmania serology and polymerase chain reaction test results were negative. The quantiferon Tb-gold staining results were negative. Malaria was excluded. Empiric treatment for sepsis was administered without improvement in the clinical condition.

Blood cultures were negative, and urine cultures revealed the growth of numerous colony-forming units of non-multidrug-resistant *Escherichia coli*.

Lumbar puncture and brain magnetic resonance imaging MR were performed considering the objectivity of ataxia, with normal findings. *Toxoplasma gondii* encephalitis was excluded due to the absence of suggestive lesions. HIV genotypic resistance testing (GRT) was performed, and on day 7th antiretroviral therapy with bictegravir, tenofovir, and emtricitabine was administered after exclusion of cryptococcosis by blood serum antigen, liquor microscopy, cultural examination, and exclusion of tubercular meningitis by polymerase chain reaction and liquor microscopy. Opportunistic infection prophylaxis with trimethoprim-sulfamethoxazole (80–400 mg daily) was administered. Subsequently, GRT did not show any resistance to antiretroviral drug classes.

**Fig. 1:**
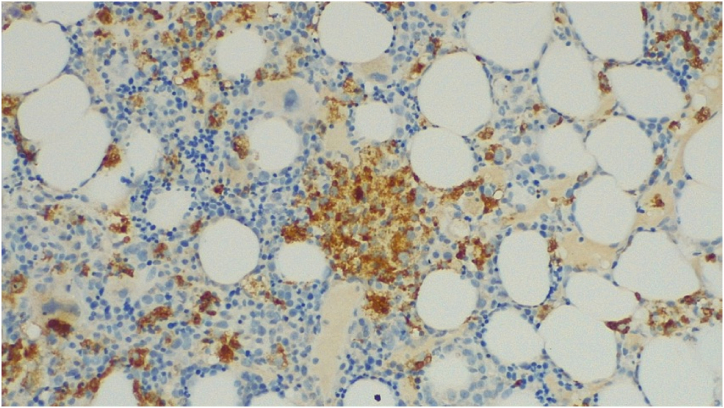
Histiocyte hyperplasia with granuloma formation (CD68 immunohistochemical staining 200x).

**Fig. 2:**
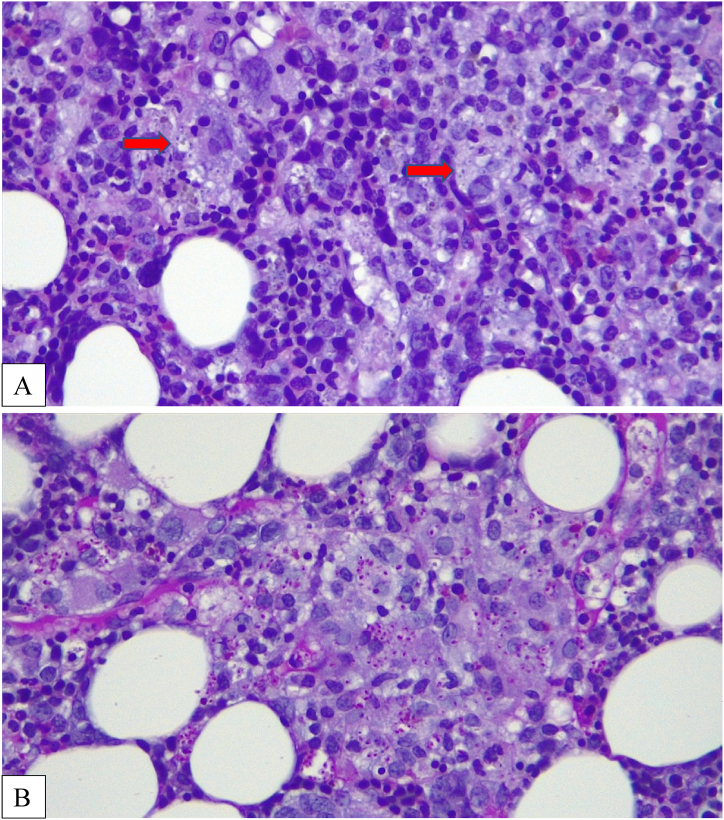
**A.** Intracellular coccoid microorganism in the cytoplasm of phagocytes (bone marrow biopsy Hematoxylin and Eosin stain × 400).**B.** Numerous intracellular organisms consistent with *Histoplasma capsulatum* (Periodic acid–Schiff stain x400).

**Fig. 3:**
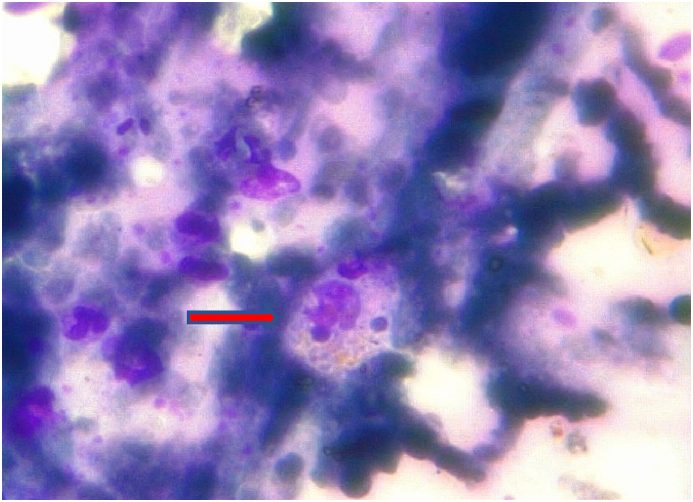
Erythroblast and coccoid inclusions suggestive of Histoplasma inside a histiocytic cell (bone marrow aspirate smear, May-Grunwald and Giemsa stain, 40x).

**Fig. 4:**
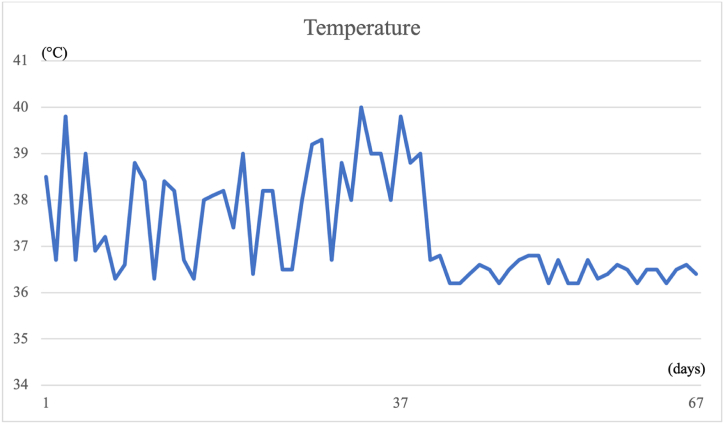
The graphic highlights the trend of body temperature during hospitalization and its modification after starting liposomal amphotericin B on the 37th day.

**Fig. 5:**
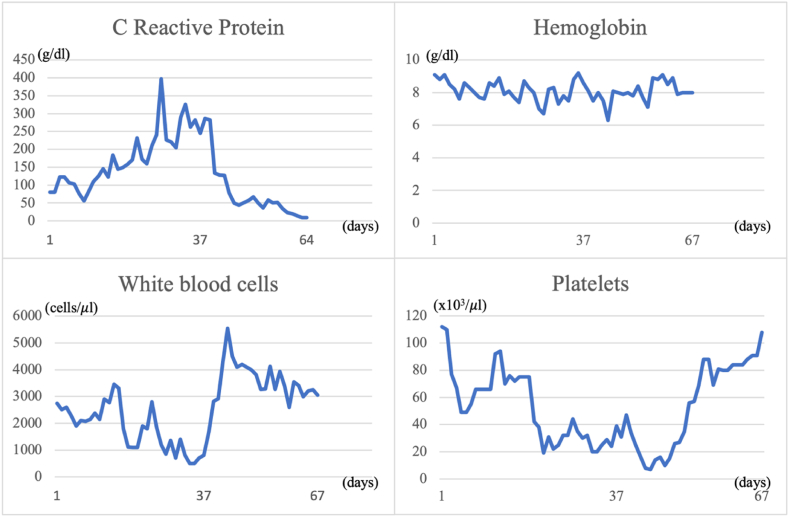
The graphic highlights the trend of blood exams (CRP, hemoglobin, white blood cells, platelets) during hospitalization and its modification after starting liposomal amphotericin B on the 37th day.

Due to the persistence of high fever, pancytopenia, elevated ferritin levels (>8,000 ng/ml), and splenomegaly, a bone marrow biopsy was performed for the suspected hemophagocytic syndrome. Microscopic examination of the bone marrow biopsy specimen revealed myelodysplasia, probably related to HIV infection, associated with focal aspects of hemophagocytosis. Corticosteroid therapy and intravenous immunoglobulin (IVIG, 1 g/kg/day for two days) were administered without improvement. On the following days, there was a clinical condition of precipitation and a significant weight loss of >10%. The patient developed psychiatric disorders characterized by psychomotor agitation, transient hallucinations and anxiety, worsening skin lesions with scattered ecchymosis, and weight loss. Subsequent blood examinations showed further platelet reduction (20 × 10^3^/μL), elevated PCT (17.9 μg/L), and persistently high ferritin (>8,000 ng/ml). Microbiological exams, including “T2Bacteria ® Panel”, blood culture and a second lumbar puncture, remained negative without microorganism growth. Another bone marrow biopsy was performed three weeks after the procedure, showing the presence of granulomas (Fig. 1), widespread hemophagocytosis, and numerous intracellular coccoids from Periodic Acid Schiff positive (Fig. 2 A, B), Ziehl-Nielsen, and Giemsa negative microorganisms. Progressive disseminated histoplasmosis with cerebral involvement was suspected, and the diagnosis was supported by positive sternal aspiration sampling (Fig. 3) and an elevated serum galactomannan index of 10.18 (<0.16). Therapy with liposomal amphotericin B (5 mg/kg per day, total dose 350 mg) was initiated, and progressive improvement of clinical conditions and blood examinations was observed, with the disappearance of psychiatric disorders and normalization of hematological parameters and inflammation markers. Fever remission was observed at 48h with a CRP and PCT levels reduced until normalization. Fig. 4, Fig. 5 show the trends in fever, hemoglobin, platelet, white blood cell, and CRP levels during hospitalization. Low-dose corticosteroid therapy was continued because the thrombocytopenia persisted. After one month, the hemoglobin level, leukocyte count, and platelet count stabilized, and steroid use was gradually tapered. Liposomal amphotericin B was administered for 31 days without any adverse reactions. The patient was switched to oral itraconazole. Itraconazole was prescribed at a dosage of 200 mg three times a day for 3 days, followed by 200 mg twice a day for a minimum of 12 months. The latest available examination results, obtained 7 months after discharge, indicated a hemoglobin level of 12.7 g/dl, a platelet count of 248 × 103/μL, and a leukocyte count of 4.58 × 103/μL. The HIV viral load was <20 copies/mL, and the CD4 cell count was 123 cells/mm3 (6.96%, CD4/CD8 = 0.1). No adverse reactions were observed during the therapy with itraconazole.

## 4 Discussion

Diagnosis of histoplasmosis is challenging in non-endemic areas because of non-specific symptoms and a lack of qualified clinical and laboratory personnel [10].

Antigen detection tests are frequently unavailable and serological testing is unreliable in immunocompromised patients. Bone marrow microscopy is the primary method of diagnosis in non-endemic areas [11], and cases of histoplasmosis are discovered accidently or following postmortem biopsies, as described in the literature [10].

Multiple organ dysfunction is common in immunocompromised patients, and the prognosis is poor without treatment. Patients may exhibit a sepsis-like syndrome characterized by disseminated intravascular coagulation, encephalopathy, acute respiratory distress syndrome, vascular collapse, and multiorgan failure.

Moreover, because of its overlapping clinical-radiological and histological characteristics, tuberculosis is the leading differential diagnosis in our geographic region, particularly in immunocompromised individuals. Comparable clinical characteristics and immunopathogenesis characterize the two disorders, and infections that have lasting dormancy for an extended period may be reawakened due to a weaker immune system [4,12,13].

Leishmaniasis, particularly cytopaenia, malaria in people coming from endemic locations, and wasting syndrome are additional pathologies that need to be considered in the differential diagnosis [[14], [15], [16]]. We believe that histoplasmosis in patients with AIDS may sometimes be misdiagnosed as wasting syndrome, leading to poor outcomes.

Haemophagocytic lymphohistiocytosis may complicate the clinical picture and make its management challenging, as in the present case. Furthermore, a barrier to the prompt identification of HLH related to disseminated histoplasmosis is the occurrence of HLH secondary to HIV infection alone, without opportunistic infection, which retards correct diagnosis [17].

Other viral infections, such as EBV and CMV, are common triggers of acquired HLH. We did not consider these as etiological agents because of the low-level viremia; therefore, no treatment with ganciclovir was needed.

We also report a recent case of SARS-CoV-2 coinfection. The patient complained of an acute presentation of fever for a few days and negative anamnesis for weight loss. Clinical deterioration happened during the hospitalization, with a reduction in hemoglobin, platelets, and white blood cells, progressive weight loss, and the appearance of new neuropsychiatric disorders such as reactivation of latent histoplasmosis had recently occurred.

We suppose that immune dysregulation related to SARS-CoV-2 viral infection triggered the reactivation of histoplasmosis and hemophagocytic syndrome, as described in another case where the patient was treated with convalescent plasma [18]. Our patient did not receive any antiviral drugs for COVID-19 or require oxygen supplementation.

The pathogenesis of HLH includes defective cytotoxic regulatory functions of macrophages due to the low activity of natural killer and Cytotoxic T cells, leading to uncontrolled hyperactivation of macrophages. As a result, cytokine overproduction occurs, leading to a cytokine storm and multiorgan failure [19].

The hemophagocytosis score (HScore) can be used to estimate an individual's risk of having reactive hemophagocytic syndrome. It is based on nine variables (known underlying immunosuppression, high temperature, organomegaly, triglyceride, ferritin, serum, AST, fibrinogen levels, cytopenia, and hemophagocytosis features on bone marrow aspirate) [20].

In our case, the number of points assigned at presentation was 206 (88–93% probability of reactive HLH), and the score increased to 256 (>99% probability of reactive HLH) at the time of the second bone marrow biopsy.

A literature search for histoplasmosis associated with HLH in HIV-positive patients yielded 33 papers describing 65 AIDS patients with histoplasmosis and reactive hemophagocytosis. The results are shown in Table 1 [10,11,18,[21], [22], [23], [24], [25], [26], [27], [28], [29], [30], [31], [32], [33], [34], [35], [36], [37], [38], [39], [40], [41], [42], [43], [44], [45], [46], [47], [48], [49]]. Sex was reported in 60 patients, of whom 45 were male. The median patient age was 42 years (IQR 32–46). Almost all cases have been reported in endemic areas, mainly South America and the United States; only 4 cases have been described in Europe, involving subjects from endemic areas, mainly Africa. All patients had AIDS with a median CD4 T-cells value of 19.5 cells/mm3 (IQR 8–37) and a median HIV-vial load of 341,097 copies/ml (IQR 231,530–1,336,194).

**Table 1:** The table shows the epidemiological and clinical characteristics of the subjects with AIDS, histoplasmosis and HLH according to the literature review.

Authors, year	Number of patients	Country of origin	Sex	Age (years)	Clinical picture	Confirmed diagnosis	Outcome
Freire et al. [10], 2022	1	South America	M	33	N and P	Yes, histology	Dead
Gupta et al. [21], 2017	1	United States of America	M	26	NS	Yes, histology and urinary antigen	Live at discharge
Castelli et al. [22], 2015	1	Mexico	M	32	NS	Yes, microbial isolation	Live at discharge
Atiyat et al. [23], 2021	1	United States of America	M	55	P	Yes, urinary antigen	Dead
Tomaino et al. [24], 2022	1	South America	M	32	NS	Yes, histology	Dead
Nieto et al. [25], 2016	1	South America	M	33	GI	Yes, histology, microbial isolation, and urinary antigen	Live at discharge
Nguyen et al. [26], 2020	12	United States of America	M (8), F (4)	46 (average age)	NS (12)	Yes (10/12), microbial isolation (10/12), histology (8/12)	Dead (2/12)
Warrren et al. [27], 2022	1	United States of America	F	42	NS	Yes, histology and urinary antigen	Live at discharge
Montenegro-Idrogo et al. [28], 2020	8	South America	M (5), F (3)	30 (average age)	GI (2), S (1), P (1), N (1), P and S (1), P and GI (2)	Yes (8/8), microbial isolation (7/8), histology (2/8)	Dead (4/8)
Subedee et al. [29], 2015	1	United States of America	F	42	NS	Yes, histology and urinary antigen	Live at discharge
Townsend et al. [30], 2015	9	United States of America (6), Mexico (1), El Salvador (1), NR (1)	M (7), F (2)	42 (average age)	P (8), NS (1)	Yes (9/9), histology (7), urinary antigen (7), and microbial isolation (8/9)	Dead (5/9)
Fogelson et al. [31], 2022	1	Honduras	M	30	P, GI, and S	Yes, histology, urinary antigen, and microbial isolation	Live at discharge
Gómez-Espejo et al. [32], 2017	1	South America	M	23	NS	Yes, histology	Live at discharge
Asanad et al. [33], 2018	1	El Salvador	M	48	NS	Yes, histology, urinary antigen, and microbial isolation	Live at discharge
Gil-Brusola et al. [34], 2007	1	South America	M	33	NS	Yes, histology and microbial isolation	Dead
Zanotti et al. [11], 2018	1	Africa	F	19	NS	Yes, histology and microbial isolation	Live at discharge
Loganantharaj et al. [35], 2018	1	Dominican Republic	M	46	NS	Yes, histology and urinary antigen	Live at discharge
González-Hernández et al. [36], 2020	1	Mexico	M	21	P and OM	Yes, histology	Live at discharge
Sanchez et al. [37], 2007	1	South America	M	61	NS	Yes, histology and microbial isolation	Live at discharge
Castejón-Hernández et al. [38], 2021	1	Africa	M	46	NS	Yes, histology	Dead
Tsuboi et al. [39], 2019	1	South America	F	56	S	Yes, histology, urinary antigen, and microbial isolation	Live at discharge
Lage et al. [40], 2022	1	South America	F	44	NS	Yes, histology and microbial isolation	Live at discharge
Ocon et al. [41], 2017	1	South America	M	49	N	Yes, histology and microbial isolation	Live at discharge
Majluf-Cruz et al. [42], 1993	3	South America	M (3)	41	NS (2), P (1)	Yes (3/3), histology (3/3) and microbial isolation (1/3)	Dead (1/3)
Touza et al. [18], 2022	1	Honduras	M	26	P	Yes, urinary antigen	Live at discharge
Guiot et al. [43], 2007	1	South America	M	43	P and GI	Yes, histology and microbial isolation	Live at discharge
De Lavaissière et al. [44], 2008	1	South America	M	33	N and OM	Yes, histology and microbial isolation	Live at discharge
Koduri et al. [45], 2022	6	United States of America	M (1), NR (5)	29 (1), NR (5)	P (2), S (1), NS (1), P and S (2)	Yes (6/6), histology (6/6) and microbial isolation (6/6)	Dead (3/6)
Chandra et al. [46], 2012	1	India	F	38	AS	Yes, histology	Live at discharge
Vaid et al. [47], 2011	1	Europe (he lived in Caribbean for many years)	M	25	P, S, and OM	Yes, histology	Dead
Kumar et al. [48], 2000	1	India	M	40	NS	Yes, histology	Dead
Chemlal et al. [49], 1997	1	Africa	M	50	S	Yes, histology and microbial isolation	NR

NR: nonreported; M: male; F: female; N: neurological involvement; P: pulmonary involvement; NS: nonspecific systemic symptoms; GI: gastrointestinal involvement; S: skin involvement; OM: oral mucous involvement.

The clinical presentation on admission was described in 37 cases, with fever, weight loss, fatigue, cough, dyspnea, and gastrointestinal disorders (diarrhea and abdominal pain) being the most common symptoms. Analyzing the cases, in 30 we identified a non-specific clinical picture without explicit organ localization; 25 presented with pulmonary involvement, 10 were mucocutaneous, 7 were gastrointestinal, and 4 were neurological. In our case, we considered neurological involvement because of the presence of ataxia and neuropsychiatric disorders, which resolved after targeted therapy.

The criteria according to the diagnostic HScore for reactive HLH [20] on admission are shown in Table 2. Anemia (Hemoglobin ≤9.2 g/dl) was the most mentioned condition; triglyceride and AST values were reported in a small number of cases, and almost all of them satisfied the criteria for reactive HLH. The mortality rate was 34% (22 deaths). Univariate analysis showed an association between a clinical picture with pulmonary involvement and mortality (odds ratio:3.6, 95% confidence interval:1.22–10.65; p, 0.02). The Mann-Whitney *U* test showed a significant difference in AST values between living and dead patients (p = 0.02), although the data were reported in only 32 cases; multivariate analysis did not confirm this result. Therefore, additional data are required to evaluate the role of transaminase levels in predicting mortality.

**Table 2:** Data on criteria of HScore according to the review of the literature.

Criteria	The number of cases in which the criterion is satisfied on the number of cases in which the data is available	Median value and IQR where applicable for all cases in which the data is available
Underlying immunosuppression	all cases	NA
Fever ≥38.4 °C	43/53	39 (38.75–39.55)
Hepatomegaly	20/43	NA
Splenomegaly	44/60	NA
Hemoglobin ≤9.2 g/dl	53/63	7.7g/dl (6.45–8.55)
White bloods cells ≤5,000/mm^3^	34/54	2,125/mm^3^ (500-3,900)
Platelets ≤110,000/mm^3^	48/60	46,000/mm^3^ (18,500–83,000)
Ferritin ≥2,000 ng/ml	44/52	16,500 ng/ml (4,850–41,489)
Triglyceride ≥132.7 mg/dl	32/34	232 mg/dl (172–363)
Fibrinogen ≤250 mg/dl	15/37	NA (value often not indicated)
AST ≥30 U/L	32/32	150U/L (100–214)
Hemophagocytosis features on bone marrow aspirate	46/54	NA

NA: not applicable.

Microbiological isolation confirmed the diagnosis of histoplasmosis in 45 cases, primarily from bone marrow samples and blood culture specimens, but also from respiratory samples and cerebrospinal fluid (CSF) in one case without neurological symptoms [45]. In our study, the cultures showed no growth, probably because of the lack of an adequate cultivation medium. CSF examination showed no atypical values and did not reveal any microorganisms, despite the presence of ataxia and neuropsychiatric disorders. Antigen detection is another commonly reported diagnostic tool, especially for urine samples. In our case, serum galactomannan was used to support the diagnosis due to the lack of specific antigen detection. Galactomannan and beta-d-glucan are the fungal cell wall components and can be detected in the serum to diagnose invasive fungal infections such as histoplasmosis [7,8]. They were not referenced in the cases we reviewed but might be helpful in supporting diagnosis in non-endemic areas where more specific tests are unavailable. In cases described in the literature, bone marrow histology was the principal tool used to confirm the diagnosis. If they are inconclusive and the clinical suspicion of reactive hemophagocytic syndrome is high, a second bone marrow examination may be helpful, as in the present case. Treatment of a patient's trigger condition is the primary treatment for reactive HLH. Antimicrobial therapy, as in the present case, is critical and can eliminate the need for chemotherapeutic medications, corticosteroids, and intravenous immunoglobulin [21]. A previous study has reported increased survival in patients with HLH secondary to histoplasmosis who received amphotericin B in combination with intravenous immunoglobulin [50].

In the series of patients described by Townsend et al. [30], all were treated with antifungal therapy, and none needed chemotherapy protocols; half of the patients received steroids, IVIG, or both without benefits. While in all cases in which antifungal therapy was not promptly administered, the patients died, confirming the lethality of the picture [23,34,42,48]. Anecdotal cases were initially refractory to treatment with amphotericin B and other medications, such as anakinra, an interleukin-1 receptor antagonist [41]. Etoposide was administered in only two cases [26,31], and corticosteroids and/or IVIG were administered in 20 patients. However, although it is recommended to treat overt inflammation of HLH with corticosteroids with or without IVIG [51] in HIV patients, we believe that prompt anti-infective therapy is the milestone for reactive HLH treatment, as documented in our case. The first-line therapy for histoplasmosis is liposomal amphotericin B, followed by oral itraconazole. Other treatment options include fluconazole and posaconazole for patients who are intolerant to first-line therapy [18,30,42,46]. Disease severity and patient immune status establish treatment duration. Patients with AIDS and disseminated disease typically require 12 months of initial therapy, followed by lifelong maintenance with itraconazole therapy to prevent relapse [52].

Liposomal amphotericin B was administered for 31 days and then switched to oral itraconazole. The persistent disappearance of fever was the first response to treatment after only 48 h, followed by a rapid and progressive reduction in inflammation indices (CRP and PCT), and finally, slow stabilization of hemoglobin, platelet, and leukocyte levels after four weeks of therapy. We believe that this case raises important issues. Given the increase in migratory fluxes from endemic countries and the high mortality risk associated with delayed diagnosis and treatment, promoting awareness of histoplasmosis in our geographic area is vital. Most cases of histoplasmosis in patients with AIDS are misdiagnosed as wasting syndrome or tuberculosis in non-endemic areas. Clinicians outside endemic areas should evaluate histoplasmosis as a cause of severe clinical picture and HLH, even when other probable causes of HLH may exist in a patient with a travel history to an endemic area, even after many years, considering the possible reactivation of latent infection.

## Declarations

### Consent for publication

Informed consent was obtained from the patient for the publication of all images, clinical data and other data included in the manuscript.

### Author contribution statement

All authors listed have significantly contributed to the investigation, development and writing of this article. </p>

### Data availability statement

Data will be made available on request.

## Declaration of competing interest

The authors declare that they have no known competing financial interests or personal relationships that could have appeared to influence the work reported in this paper
